# Supplementary figures and images for: Ecological and anthropogenic factors influencing the Summer habitat use of Bos gaurus and its conservation threats in Chitwan National Park, Nepal
Source: PeerJ. 2024 Sep 20;12:e18035. doi: 10.7717/peerj.18035 (PMC11418812; doi:10.7717/peerj.18035)

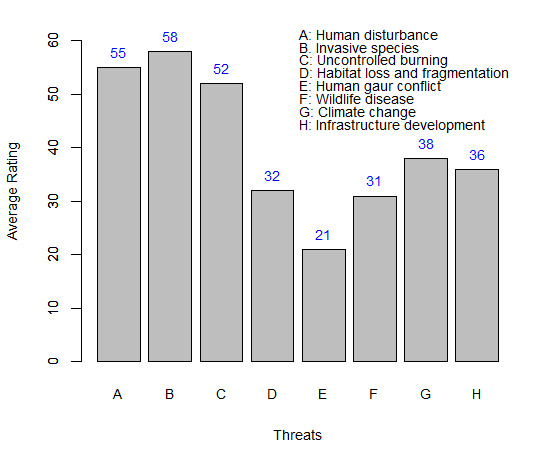

Supplement: Supplemental Information 1 — The average rating assigned to each threat, with emphasis on selecting the top five threats for the final ranking procedure. [file peerj-12-18035-s001.jpg]
